# Cognitive bias toward the Internet: The causes of adolescents’ Internet addiction under parents’ self-affirmation consciousness

**DOI:** 10.3389/fpsyg.2022.891473

**Published:** 2022-08-01

**Authors:** Mindan Zhou, Jianfei Zhu, Zhibo Zhou, Huiqi Zhou, Guoping Ji

**Affiliations:** ^1^School of Marxism, Jilin University, Changchun, China; ^2^Nanchang Institute of Technology, Nanchang, China; ^3^School of Economics and Management, East China Jiaotong University, Nanchang, China; ^4^School of Humanities, Jiangxi University of Finance and Economics, Nanchang, China

**Keywords:** adolescent Internet addiction, cognitive bias, self-affirmation, empathy, Internet

## Abstract

The Internet plays a crucial part in the adolescent life. However, as a product of modernization, the Internet has brought a lifestyle different from that of our parents who tend to regard excessive exposure to the Internet as a manifestation of the adolescent Internet addiction. The cognitive bias against the Internet seem to have been arisen among the parents. Under the theoretical framework of self-efficacy and empathy, this study adopts PLS-SEM to analyze the contributing factors of the adolescent Internet addiction from the perspective of self-affirmation consciousness of parents. The result demonstrates that self-affirmation consciousness has a significant positive effect on the empathy process; the empathy process and self-affirmation have a significant positive effect on cognitive bias; and the empathy process acts as a mediator between self-affirmation and cognitive bias. To sum up, through the investigation of the causes of adolescent Internet addiction, this study explores the formation process of parents’ cognitive bias toward the Internet under the influence of self-affirmation consciousness, verifying the practical effects of empathy in the process of promoting rational thinking of parents toward the Internet and adolescent Internet use, and at the same time promoting the harmonious development of parent–child relationships to a certain extent.

## Introduction

In recent years, with the innovative development of the Internet and information technology, information products such as digital media and social platforms are constantly emerging and rapidly being upgraded. Under the international trend that countries around the world are committed to technological development to enhance national competitiveness with information as the core. The improvement of Internet infrastructure construction and further popularization mobile devices are of great importance to strengthen the penetration of digital communication into the public society and increase the power of digital culture (Dunne-Howrie, 2022; Geng et al., 2022). While the fast development of the Internet has changed the course of human civilization and promoted social revolution. And the virtual private space provided by the Internet for human beings promotes the development of real modern society to a large extent (Gong et al., 2019; Wu et al., 2020). Meanwhile, it provides infinite possibilities for the growth of human collective consciousness. Human society has been continuously presented to people through the Internet in a more varied form, which has greatly enriched the human civilization (Yi et al., 2021, 2022). The Internet has evolved over the past 25 years, in which social media, characterized by massive information interaction, has rapidly risen to occupy most of people’s social interactions. As the Internet is getting increasingly closer to people’s life, and the adolescents are constantly integrating with the Internet world in a new way as an indispensable user group (Zhou and Fang, 2015).

Currently, the Internet has highly integrated into all aspects of the adolescent growth. Along with their study, daily life, entertainment and social interaction, it has become the main channel for the adolescents to understand the world and become the source of knowledge for them to form values. However, with the continuous popularization of the Internet and social tools such as computers, tablets, and smart phones, the adolescent’s focus of life shifting to the Internet has become a trend whereas it has also brought thorny problems and the Internet addiction is one of them (Li et al., 2022, 2021). As a typical label for excessive use of the Internet, the Internet addiction was first proposed and named by American scholars Kimberly and Young in 1996 after referring to the Substance Dependence Disorders (DSM) IV Criteria in the *Diagnostic and Statistical Manual of Mental Disorders* (Young, 1996). Besides, Griffiths also published case studies on the male and female Internet addiction in 1996. And they are the pioneers of the Internet addiction research (Griffiths, 1996). Due to the prominent social function of the Internet, the Internet addiction has also been defined as an instrumental form of social interaction (Cerniglia et al., 2017; Gong et al., 2020). Contemporary adolescents are the greatest beneficiaries of the Internet popularization, and the prevalence of the Internet addiction among adolescents has gradually emerged. Consequently, the characteristics of the adolescent Internet use and the effects of widely used social media on their development have become valuable research topics.

In terms of the external influences on adolescent Internet addiction, scholars currently focus on psychological factors, such as personality traits (Young and Rogers, 1998); demographic factors, such as gender (Ko et al., 2005); social factors, such as social support (Hardie and Tee, 2007), family economic status (Hur, 2006), etc. In addition, a great number of studies have shown that poor parent–child relationships have a vital important impact on adolescent Internet addiction; harmonious parent–child relationships can effectively reduce the frequency of adolescents’ Internet use, while parent–child conflict increases the likelihood of Internet addiction (Luqman et al., 2020; Wei et al., 2020; Huang et al., 2021). How to help adolescents become masters of cyberspace rather than captives of the Internet is an educational theme that every parent has to face in an era of instant information. However, due to the gaps in information reception, knowledge level, and awareness, parents have different degrees of cognitive bias toward the Internet and their children’s use of it. The root of solidified thinking is their self-awareness catalysis, and when their children’s behavior conflicts with their own perceptions, parents naturally act to block it due to their cognitive biases, so it can be said that parents’ cognitive bias toward the Internet is the result of self-affirmation. Available research has explored many factors influencing adolescent Internet addiction and its development, but little applied research has been conducted in conjunction with psychology. The parental cognitive bias toward the Internet is a product of self-affirmation, and the causes of adolescent Internet addiction need to be explored under this perspective of empathy. In this study, we will explore how self-affirmation influences the formation of parental cognitive bias toward the Internet and verify whether this process is mediated by empathy, to systematically understand the mechanism of adolescent Internet addiction.

## Literature review

### General self-efficacy and self-affirmation

Proposed by psychologist Bandura, self-efficacy is defined as the extent or strength of one’s own ability to complete a task in a specific situation (Bandura, 1977). As a core variable in the individual self-belief system, self-efficacy is an important approach for individuals to deal with traumas (Feeney and Collins, 2014). Self-efficacy plays a major role in the self-regulatory system. The individuals’ perception of their own abilities affects their choice of activities, and such perception could also be a conscious manifestation of self-affirmation in many cases. First proposed by Steele, self-affirmation means that when individuals encounter external threats, they will re-evaluate their self-worth to maintain their integrity and security. Such self-affirmation aims to alleviate the negative effect of threats on individuals (Steele, 1988). Generally, the effects of self-affirmation are constrained by two conditions, specifically, self-affirmation is effective only for threatened individuals itself and it has no effect on other defense mechanism that already functioning (Chen H. C. et al., 2020; Easterbrook et al., 2021; Zhang et al., 2021). The self-affirmation theory holds that when an event or information that threatens self-integrity appears, the individual will activate the self-defense mechanism and reallocate cognitive resources to event processing. Limited by cognitive resources, a sense of self-affirmation hinders individuals’ objective and unbiased perceptions of people or events (Sherman and Cohen, 2006; Khalid et al., 2021). This effect is particularly evident in the elder generation’s perception of the Internet. Under the traditional self-knowledge system, the elder generation have certain prejudices about the Internet, and when adolescents use the Internet parents will resort to criticism or confiscation of devices, etc. to restrict their use of the Internet, and even expect such actions to forcibly sever the connection between their children and the cyberspace, yet it is this kind of cognitive bias that stiffens the parent–child relationship (Luqman et al., 2017; Hsieh et al., 2020).

From a macro perspective, the scope of research and application of the self-affirmation theory engagement is constantly extending, while from a micro perspective, the self-affirmation theory can also be seen as an extension of the empathy theory research. Existing studies have explored the impacts of the self-affirmation theory on interpersonal relationships from the perspective of self-cognitive value (Hurst et al., 2020); besides, researchers have verified a positive relationship between the self-affirmation theory and pro-environmental behavior (Graham-Rowe et al., 2019). Studies on self-affirmation and empathy have shown that empathic processing is actually a concern for oneself, and that the effect of self-affirmation on empathy causes people to show more positive emotions and strength (Exline and Zell, 2009); another study on empathy also showed that self-affirmation affects the likelihood of paying attention to and helping others out of empathy (Ministero et al., 2018); emotional and cognitive errors arising in multicultural contexts shape people’s risk prevention and control behavior, which is guided by empathic factors to make choices out of self-protection and self-affirmation (Li and Katsumata, 2020; Yi et al., 2022). Based on the above background analysis, to clarify the research direction of the formation of adolescent Internet addiction, this study will introduce empathy theory as a potential theoretical support, in order to explore the inner relationship between self-affirmation theory, empathy process and cognitive bias, so as to systematically analyze the formation mechanism of adolescent Internet addiction.

There are varied opinions on the effects of self-affirmation and empathy. Empathy is an investigative tool used by individuals to gather emotional information through cognitive processes and emotion simulation (Davis, 1983). The current stage of research on empathy focuses on three dimensions: emotional empathy, cognitive empathy, and affective bias. Specifically, emotional empathy refers to the ability to generate spontaneous alternative emotional experiences to others’ emotional feelings; cognitive empathy refers to the ability to recognize others’ emotions and understand others’ perspectives; and affective bias refers to the deviation of cognitive outcomes from objective reality due to the influence of internal or external factors when perceiving oneself, others or environment (Vachon and Lynam, 2016). First, in the research on emotion regulation and positive behavior change through self-affirmation, it is believed that self-affirmation can facilitate individuals to reduce social anxiety symptoms and inspire individuals to achieve higher positive emotions (Lakuta, 2020); the case study on smoking has found that compared with the control group in the behavior of smoking, the smokers with positive self-value gained more positive emotions and increased their own receptivity to relevant information as well (Crocker et al., 2008). This demonstrates that self-affirmation affects individual emotions and moods. From a cognitive perspective, self-affirmation improves the efficiency of self-control, in other words, self-affirmation changes the focus of individual attention to the value of information itself in the process of changing self-recognition (Davis et al., 2016; Wang et al., 2020a). For instance, in the survey of how caffeine cause health problems, compared with the control group, the participants with self-affirmation had weaker defensive responses to the threat information and they were more efficient in information collection and extraction, which facilitated their integration of information (DiBello et al., 2015). In this era of information explosion, emotional fluctuation is an important driving force in the evolution of online public opinion. Parents could be easily influenced by objective factors such as opinion leaders or subjective factors such as herd mentality due to differences in self-affirmation and awareness of social phenomena, resulting in affective bias in the reception and feedback of information (Peters et al., 2014; Duan et al., 2022). In addition, parents’ self-affirmation often drives a series of psychological activities such as emotional empathy and cognitive biases in the parent–child relationship (Huang et al., 2021; Masood et al., 2022). Accordingly, this study proposes the following research hypotheses:

***H1a:** The greater the sense of parental self-affirmation, the greater the occurrence of their emotional empathy.*
***H1b:** The greater the sense of parental self-affirmation, the greater the occurrence of their cognitive empathy.*
***H1c:** The greater the parental self-affirmation, the greater the occurrence of their emotional deviance.*

### Parent–child empathy relationship

#### Blind trust derived from family affection

The word empathy was first translated from German by a psychologist Edward Titchener in 1909. Due to the rapid development of information transmission, the concept of empathy has become a hot academic topic and it is widely used in disciplines such as medicine, psychology, journalism and communication, and tourism (Kupetz, 2020; Yi et al., 2022; Wang et al., 2022). The transference has been widely applied in researched related to the intergenerational relationships between the elder and younger generations. Positive parent–child relationships and effective communication not only contribute to the normal functioning of the family but also to the development of adolescents, alleviating their discomfort during socialization (Tamarit et al., 2021; Uzun et al., 2021). Most of the existing studies have explored the kinship between parents and children from the phenomenon of left-behind children, focusing on the impact of intimate relationships on children’s sense of security, well-being and psychological health (Niu et al., 2020; Li et al., 2022). With the continuous development of the Internet, communication between parents and children has gradually shifted from face-to-face to the virtual world, which brings a trust crisis in the parent–child relationship, and the conflict between parents’ resistance to the Internet and children’s use of the Internet destroys family harmony, and the Internet addiction of adolescents is thus aggravated (Wang et al., 2020b; Qiu et al., 2022). And this negative impact from the crisis of trust, in turn, deepens the parents’ cognitive bias toward the Internet (Steinberg et al., 2021). Blind trust between parents and children arising from intimacy may also pose a potential threat to the intimate relationship between the two. When emotional empathy occurs, parents may have varying degrees of cognitive bias toward the Internet due to their adolescent’s Internet addiction (Tian, 2016). Based on the above, the following hypotheses are proposed.

***H2a:** Parental emotional empathy impacts the emergence of cognitive biases.*

#### Dialectical rational thinking

The Core Information and Interpretation of the Chinese Adolescent Health Education (2018 edition) defines the Internet addiction (IA) as the uncontrollable behavior of the urges to use the Internet under the influence of non-addictive substances, which manifests as excessive exposure to the Internet leading to obvious impairment of academic and occupational performance and social function. Addiction is a mental illness. Addictions in the traditional sense is very similar to excessive smartphone use, but they also could be distinctly different. Excessive cell phone use is a behavior, whereas addiction is a psychiatric disorder that is detrimental to physical and mental health. Extant research does not find a clear basis for smartphone dependence in terms of addiction, and although this behavior is similar to a psychiatric disorder, the two cannot be fully equated (Panova and Carbonell, 2018). Strictly speaking, Internet addiction may only be an exaggerated expression of excessive Internet use. From an objective point of view, Internet socialization and video games are necessary for adolescents’ daily interactions; the Internet is not only an assistant for adolescents’ learning but also an important link with the outside world, and it can help adolescents escape from the stress and pain brought by the real world to a certain extent for a short period of time (Masood et al., 2020; Tamarit et al., 2021). However, propelled by the public opinion, the Internet addiction has gradually become a concept constructed by parents to control adolescents’ excessive exposure to the Internet and justified the parental intervention on the Internet. In studies on cyber instruction, rational communication between parents and adolescents can moderate the sensitive relationship between cyber instruction and adolescent Internet addiction. On the one hand, rational communication effectively reduces the sensitivity of adolescents to Internet addiction; on the other hand, irrational communication leads to cognitive and emotional biases in adolescents (Xin et al., 2021). A survey of parents and children showed that although more than half of the parents believed that the content of the Internet was positive, they still classified the Internet as a forbidden place on account of its effect on learning because the Internet was consciously viewed harmful, and such perception has been continuously solidified (Kuss and Griffiths, 2017; Wan et al., 2021). In conclusion, parents’ unscientific perceptions continue to solidify under the group effect, giving rise to a bias against the Internet that can only be addressed by rational thinking with a dialectical perspective. Based on this, the study proposes the following hypotheses.

***H2b:** Parental cognitive empathy impacts the emergence of cognitive biases.*

#### Egoistic habit

Egoism firstly mentioned in Plato’s *The Republic*, the word that originated from the Latin ego, and then under the baptism of the Renaissance and the Enlightenment, underwent a long development and gradually deriving its meaning into man’s desire and pursuit of humanity and human rights (Knez, 2016). Steiner in *The Ego and His Own* talks about the development of the individual as ending with the rationalization of egoism, meaning that egoism is the ultimate and fundamental purpose of human behavior (Clulow, 2016). Furthermore, Egoism regards the pursuit of self-interest as the nature of human beings. Thomas Hobbes once argued that individual behaviors are driven by personal interests, which take precedence over all life attitude and code of conduct (Machan, 2016). Generally, there are two types of explanation of egoism in the academic circle. One is psychological egoism, meaning that the individuals always do what is the most congruent with their own interests; and the other is ethical egoism, implying that the individuals put themselves on a commanding height of moral life when performing unjustly (Vries et al., 2010). Today, thought patterns are being impacted by the market economy, and refined egoism – the satisfaction of personal interests in a more advanced and hidden way – has become a new trend of thought. With the increasingly close relationship between the adolescents and the Internet, the egoistic thinking of the parents has gradually emerged in their views on the Internet and the children’s education. On the one hand, the adolescent Internet addiction is closely related to the educational approach of the parents. According to the above logic, it makes sense that people will always maximize their own interests.

For parents, it is based on their own cognition of the Internet to reduce their children’s exposure to the Internet to conform to their own expectation (Setiawati et al., 2021). Parents will always stand on the moral high ground to control their children’s thoughts and restrict their behavior from the perspective of maximizing their interests, which is inertial egoism at work. Not only that, under the condition of egoistic inertia, parents will have cognitive and emotional biases toward the behavior of their adolescents on the Internet, which means that the emotional input of parents changed (Avirbach et al., 2019), thus affecting the perception of the Internet in the elder generation. When adolescents become overly dependent on the Internet and reduce communication with their parents, those series of acts lead to parents’ subjectively and emotionally categorize the Internet as an undesirable factor, which results in their cognitive bias toward the Internet. Based on this, the following hypotheses were developed.

***H2c:** Parental affective bias impacts the emergence of cognitive bias.*

### Cognitive biases in the psychological empathy perspective

The term cognitive bias originated in psychology and was later incorporated into behavioral economics, and scholars have mostly adopted a limited rationality perspective when exploring cognitive bias, as Nobel Prize winners Kahnema and Tversky held a view that the brain thinking may be affected by a variety of unconscious biases due to the bounded rationality of individuals, namely cognitive biases (Tversky and Kahneman, 1974). Behavioral psychologists have found through extensive experimental investigations that actual decisions made by humans under conditions of uncertainty deviate from the predicted behavior of expected utility theory, and that people in decision making tend to exhibit limited rationality, which is known as cognitive bias (Hertel and Mathews, 2011). There are various human cognitive biases, some of them are transient and could trigger more efficient actions at the moment; some of them may cause perception biases due to individuals’ limited information processing ability and their incompleteness of information, which may affect individuals’ decision-making (Norman, 2014; Landucci and Lamperti, 2021; Xu et al., 2021). Cognitive bias is a subjective feeling of individuals affected by information. In the extant literature, studies on irrational biases such as optimism, jealousy, and narcissism are favored by scholars in various disciplines (Marshall et al., 2013). It follows that cognitive biases are actually generated by a combination of human perception and psychological drives.

As psychology continues to be applied in research related to intimate relationships, empathy has also emerged as an important lens for exploring the characteristics of parent–child relationships and as a key factor in triggering certain attitudes and behaviors (Tillery et al., 2020). It has been shown that empathy is an important component in the generation of identity and attachment, which helps humans understand others’ perspectives, needs, and intentions, and it also enhances mutual trust (Yi et al., 2021). The formation of cognitive bias toward the Internet in the elder generation is inseparable from the individual’s identification with the group concept, and in the Internet era, the individual’s sense of self-affirmation is reinforced through the reception and understanding of online information, which resulting in prejudice toward the Internet (Balakrishnan and Griffiths, 2018). In the early stages of this cognitive bias formation, the stimulus response generated by psychological empathy occupies a pivotal position. And the individual develops an imbalanced perception of the absent psychological factors of the existing situation and thus seeks self-regulatory recovery (Velvet, 2020). In family life, self-affirmation is inseparable from parents’ empathic understanding of the Internet itself and their children’s use of it. Effective psychological empathy can correct parents’ one-sided perceptions of the Internet, which raises concerns about the mechanism of the role of empathy as a psychological transition variable. Meanwhile, it has been argued that when parents feel that the information, they have is more valuable than the facts, their behavior will be influenced by a sense of self-affirmation and affect their own value judgments about adolescent Internet addiction through mediating factors such as emotion, cognition, and mood (Kumar and Goyal, 2015; Wang and Yi, 2020; Zhang et al., 2021). As addressed above, empathy is a mediating mechanism in the process of driving behavioral choices and is also a mediating factor in the creation of a sense of self-affirmation and parental cognitive bias toward the Internet. Therefore, the following research hypothesis is proposed.

***H3a:** Parental cognitive biases toward the Internet are mediated by emotional empathy.*
***H3b:** Parental cognitive bias toward the Internet is mediated by cognitive empathy.*
***H3c:** Parental cognitive bias toward the Internet is mediated by emotional bias.*

### Parent–child relationship and affective interaction

**FIGURE 1:**
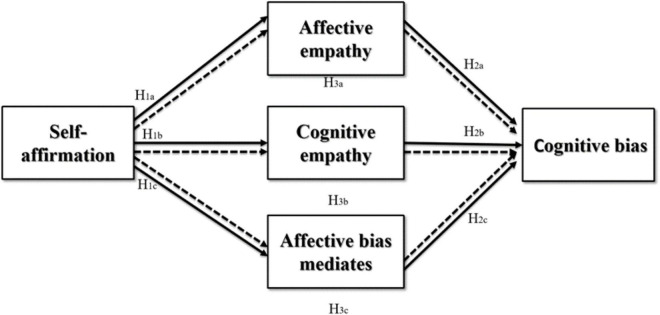
Research hypotheses model.

The ecosystem theory proposed by Bronfenbrenner (1986) argues that family is an integral part of the ecosystem and plays a key role in the development of the individual. Family is the first communication environment that an individual has to deal with, and a sound parent–child relationship is the vital foundation for individual’ development and is conducive to the improvement of personal resilience. Previous studies on the parent–child relationship and traditional bullying have found that parental affect can predict whether their children will be bullied, and the parent–child conflict is positively correlated with behaviors of being bullied (Boniel-Nissim and Sasson, 2018). As the Internet has become an indispensable part today, the impact of the parent–child relationship on the adolescent behavior has also migrated from the real world to the virtual world. Studies have shown that individuals with a harmonious parent–child relationship have better Internet literacy, and sound parent–child attachment can inhibit the formation of the Internet addiction in adolescents to a certain degree (Yusuf et al., 2020). Based on previous studies in the academic community, it is revealed that the parent–child relationship has long been understood as one-way influence relationship of parents on their children (Yoo et al., 2014), but in fact, the parent–child relationship is a friendly interaction model between parents and children. It includes not only the care and love of the parents for their children, but also the gratitude and respect of the children to their parents. Moreover, it is also a concentrated expression of the harmonious and warm atmosphere of the family (Trumello et al., 2021). A sound parent–child relationship is the manifestation of deep emotional interaction between the elder and younger generations. The parents can negotiate with the children about the time they use the Internet to prevent them from being addicted to it. In addition, a sound parent–child relationship can also implicitly shape correct values and ideological qualities of young people, so that they can stay awake and handle harms calmly when they are exposed to Internet abuse. Based on the above considerations, the following research model is formed (as shown in Figure 1).

## Research design

### Respondents and procedure

In the information age, the relationship between adolescents and the Internet has become increasingly close. Based on the rapid development of the Internet in China, this study investigates the close connection between adolescents and the Internet. The development trend in China shows that the Internet has gradually integrated into all aspects of adolescents’ education, life, socialization, and entertainment. The Internet has become a major channel for adolescents to understand the world and a source of knowledge for their value formation. In contrast, parents also hold mostly negative attitudinal tendencies toward their children’s Internet use. To further explore the causes of Internet addiction among domestic adolescents and parents’ attitudes toward adolescents’ Internet use, we conducted a pre-study and a formal study with domestic parents as survey respondents.

Before the formal research, a pre-survey of parents has been conducted through field surveys and online distribution of questionnaires, and 121 valid questionnaires were collected in the pre-survey stage by screening out invalid ones. The PLS-SEM model was applied to analyze the samples of the pre-study. In terms of reliability and validity of the model, the current study adopted standardized results for analysis, as shown in Table 1. As for self-affirmation, the Cronbach’s Alpha was 0.862, rho_*A* was 0.864, composite reliability (CR) was 0.901, and average extracted variance (AVE) was 0.646; as for cognitive empathy, the Cronbach’s Alpha was 0.915, rho_*A* was 0.921, CR was 0.932, and AVE was 0.663; as for affective empathy, the Cronbach’s Alpha was 0.868, rho_*A* was 0.870, CR was 0.901, and AVE was 0.603; as for affective bias, the Cronbach’s Alpha was 0.880, rho_*A* was 01.319, CR was 0.853, and AVE was 0.540; as for cognitive bias, the Cronbach’s Alpha was 0.816, rho_*A* was 0.816, CR was 0.878, and AVE was 0.644. In the model testing process, the *R*^2^ value of cognitive bias was 0.663, and the adjusted *R*^2^ value was 0.656, indicating that each latent variable had a strong explanatory power for cognitive bias. Also, the Cronbach’s Alpha coefficient of each latent variable in the study was greater than 0.8, indicating that each latent variable had high reliability; the CR was greater than 0.7, further proving that the reliability of the model is high; and the AVE of each latent variable was greater than 0.5. In addition, the GOF value of the pre-study model was 0.46 (greater than 0.36) obtained by the formula of GOF value, which proves the goodness of fit of the model. The above analysis showed that the overall goodness of fit of the model is excellent, the internal latent relationship has a significant explanatory power, the estimated effect is acceptable, and the reliability of each variable is consistent with the construct validity.

**TABLE 1:** Construct reliability and validity.

	Cronbach’s alpha	rho_*A*	Composite reliability	Average variance extracted (AVE)
Self-affirmation	0.862	0.864	0.901	0.646
Cognitive empathy	0.915	0.921	0.932	0.663
Affective empathy	0.868	0.870	0.901	0.603
Affective bias	0.880	1.319	0.853	0.540
Cognitive bias	0.816	0.816	0.878	0.644

**FIGURE 2:**
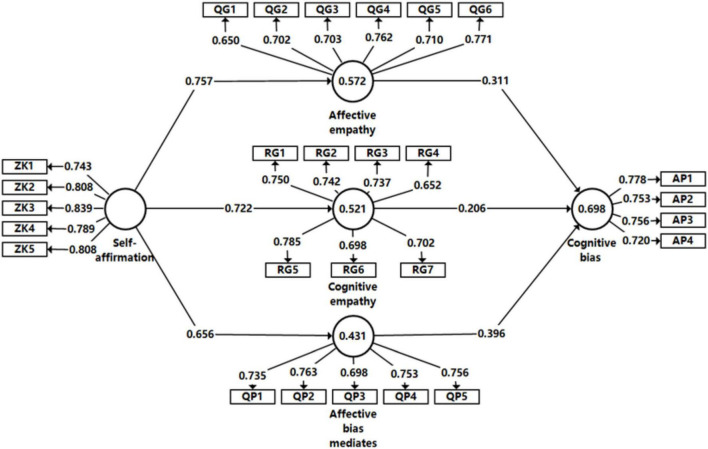
Measurement model diagram.

The formal survey was carried out on the basis of the pre-survey (see Figure 2). The formal survey included online and offline questionnaires. A total of 459 questionnaires were collected, including 287 online and 172 offline questionnaires. To ensure the validity of the questionnaire sources, the invalid questionnaires were eliminated such as those filled in within 1 min, with too many missing data, with inconsistent options. Finally, 407 valid questionnaires were obtained, with the effective rate being 88.6%, which met the research requirements, and the basic information of the samples is shown in Table 2 below. The demographic information of the interviewed parents revealed that in terms of gender ratio, the respondents included 177 males and 230 females, respectively, accounting for 43.5 and 56.5% of the total, thus the gender ratio was roughly balanced; in terms of age, the respondents aged 30–39 years old accounted for the largest proportion, at 43.5%; in terms of occupation, the self-employed and related practitioners accounted for the largest proportion; in terms of study experiences, the respondents with a bachelor’s degree or a high school background accounted for largest proportion reaching 80.6%. The study applied the PLS-SEM model to verify the research hypotheses from the perspective of prediction.

**TABLE 2:** Descriptive statistical analysis.

Item	Demographic characteristics	Number	Percentage
Gender	Male	177	43.50%
	Female	230	56.50%
Age	Below 30	145	35.60%
	30–39	177	43.50%
	40–50	77	18.90%
	Above 50	8	2.00%
Occupation	Corporate employees	87	21.40%
	Party and government personnel	66	16.20%
	Public institution employees	109	26.80%
	Self-employed	77	18.90%
	Others	68	16.70%
Educational background	Junior high school and below	37	9.10%
	Senior high school	76	18.70%
	Undergraduate	252	61.90%
	Postgraduate or above	42	10.30%
Item	Demographic characteristics	Number	Percentage
Gender	Male	177	43.50%

### Scale selection

According to the research objectives, the parent–child empathy relationship involved in this study can be measured by the Affective and Cognitive Measure of Empathy (ACME) (Vachon and Lynam, 2016), which is superior to other empathy measures in terms of measurement accuracy and excellence; besides, the current study selected the General Self-Efficacy Scale (GSES) as a reference on account of the essential properties of self-efficacy; The scale items related to “cognitive bias” in the questionnaire were designed and adapted based on the cognitive bias dimensions in the Davos Assessment of Cognitive Biases Scale (DACOBS) (van der Gaag et al., 2013). The scales for the empirical analysis in this study are shown in Table 3.

**TABLE 3:** Scale items of adolescent Internet addiction.

Study scale	Items’ tags	Measurement question items
Self-affirmation	ZK1	I can solve the problem if I try my best.
	ZK2	It is easy for me to stick to my ideal and reach my goal.
	ZK3	I am confident that I can deal effectively with anything unexpected.
	ZK4	I trust I can handle the problem and face the difficulty calmly.
	ZK5	When faced with problems, I can usually find several solutions.
Affective empathy	QG1	I’m happy when my child needs my help.
	QG2	When I think what I do is beneficial to my child, I will not worry much whether the relationship between my child and me will be affected.
	QG3	When I think what I do is good for my child, I will not be affected by the opinions of others.
	QG4	I think in most cases I can understand what my child thinks and does.
	QG5	When I notice that what I do causes negative emotion in my child, I will stop immediately.
	QG6	When my children are perplexed and upset, I often try my best to make them feel better.
Cognitive empathy	RG1	I can figure out the circumstances when the child is scared.
	RG2	I can tell at a glance when the child pretends to be happy.
	RG3	I can generally understand why the child thinks this way about something.
	RG4	I think it hard to figure out how the child feels about something.
	RG5	I can generally predict the feelings of the child when something happens.
	RG6	When the child gets angry, I can usually guess the reasons.
	RG7	When the children are sad, I can notice the sadness from their faces.
Affective bias	QP1	I am delighted with a sense of accomplishment when the child succeeds after following my arrangement and instruction.
	QP2	I am frustrated when my child does not follow my instruction.
	QP3	I hope the children can understand and recognize my care and efforts to them.
	QP4	I hope my child can behave as I expect.
	QP5	Since I’d not like to be thought irrational by the children, I will stop my action that they are against.
Cognitive bias	AP1	I find that the children use the Internet more for entertainment (like games).
	AP2	I don’t understand why the children are so dependent on the Internet.
	AP3	When the children use the Internet, I can’t help paying attention to what they are doing.
	AP4	In order to ensure children have a safer environment for their growing up, I will be wary of their exposure to the Internet.

## Empirical research

In the past research, the paradigm of constructing structural equation models by domestic and foreign scholars is relatively common, mostly through the structure of the covariance matrix of observed variables as the modeling computational logic, to construct CB-SEM (Covariance base structural equation model) among many latent variable conformations. The CB-SEM technique enable us to detect theoretical. The CB-SEM technique enable to detect the intrinsic structural relationships of concepts, but is extremely demanding in terms of model fitness, so it is more suitable for testing purely theoretical models (Gefen et al., 2011). A study by Hair and other scholars confirmed that when the sample size is too small in the structural equation model construction process and the data cannot satisfy the standard normal distribution, the CB-SEM technique is unable to test the correlation between variables from the prediction perspective It is difficult to form a complete construction of the structural equation model. In order to properly address the many limitations of CB-SEM for the non-pure theoretical model testing process, the PLS-SEM (partial lease square structural equation model), which focuses on the study of the main structure of the variables rather than the overall model construction superiority, is used instead based on the least square estimation method. This model mainly constructs the relationship between the observed and latent variables through a system of linear equations, which is basically a generalized general linear model. It has been widely used by domestic and foreign scholars in recent years because of its ability to effectively solve the covariance problem between observed variables and reduce the impact of regression unhelpful noise (Sarstedt et al., 2020). Therefore, Smart PLS is applied for data analysis in this study.

### Reliability and validity analysis of structural plane

The *R*^2^ values of the four variables of cognitive empathy, affective empathy, affective bias, and cognitive bias in Table 4 are 0.521, 0.572, 0.531, and 0.698, all of which are greater than 0.5, indicating that the interpretability of each latent variable is strong. Meanwhile, the Cronbach’s Alpha coefficient of each latent variable was greater than 0.7, indicating that each latent variable had good reliability and internal consistency. The combined reliability CR of each latent variable met the requirement of greater than 0.7, which further proved the high reliability of the model. The average extracted variance and rho_*A* of each latent variable are close to or greater than 0.5, both of which reach the evaluation criteria of PLS-SEM modeling proposed by Hair’s team (Hair et al., 2019). In addition, *Q*^2^ is a statistic that assesses the influence of exogenous variables on endogenous variables. The *Q*^2^ values of the four variables of cognitive empathy, affective empathy, affective bias, and cognitive bias in the model are all close to 0.25, which meets the criteria, indicating that the exogenous variables have a greater influence on the endogenous variables, which means that the model has a stronger predictive relevance. The *Q*^2^ value of cognitive bias in Table 4 is 0.285 greater than 0.25, which indicates that the exogenous variables of the model have stronger predictive relevance to the endogenous variable of comprehensive development level, indicating that the predictive capability of the PLS model as a whole is strong. The GOF value is 0.43 according to the calculation formula, which indicates $G\,O\,F=\sqrt{C\,o\,m\,m\,u\,n\,a\,l\,i\,t\,y}\times \bar{R^{2}}$ that the model has a strong goodness of fit. After the calculation is completed, the SRMR value is 0.074 (less than 0.08) according to the fitting results of SmartPLS, which indicates that the model has a good degree of fit overall.

**TABLE 4:** Construct reliability and validity.

	Cronbach’s alpha	rho_*A*	Composite reliability	Average variance extracted (AVE)	*R* ^2^	*Q* ^2^
Self-affirmation	0.857	0.859	0.898	0.637	–	–
Cognitive empathy	0.849	0.849	0.886	0.526	0.521	0.186
Affective empathy	0.811	0.814	0.864	0.515	0.572	0.279
Affective bias	0.795	0.795	0.859	0.549	0.531	0.235
Cognitive bias	0.744	0.743	0.839	0.566	0.698	0.285

### Loads analysis and collinearity analysis of model factor

To further explore factor loadings and collinearity characteristics of the model and ensure the reasonableness of the internal and external structure of the formative measurement model, the study calculated the model factor loadings and collinear VIF values. As can be seen from Table 5, in terms of factor loadings, all 20 level 5 indicators exhibit high polarity after orthogonal rotation, which indicates that the data structure is consistent with the model expectations. PLS-SEM is essentially a generalized general linear model, the collinear VIF value of each indicator needs to be measured, and the higher the VIF value, the higher the level of collinearity of the model. If the VIF value is less than 3, the collinearity of each indicator of the formative measurement model to be constructed is extremely low and at the ideal level and can be analyzed subsequently. The VIF values of all indicators of the PLS-SEM model constructed in the study are less than 3, which basically meet the criteria of model collinearity criterion proposed by Hair’s team, so the path coefficient analysis of PLS-SEM can be performed.

**TABLE 5:** Model factor loads and collinear VIF values.

	Cognitive bias	Affective empathy	Affective bias	Cognitive empathy	Self-affirmation	VIF
AP1	0.778					1.548
AP2	0.753					1.520
AP3	0.756					1.419
AP4	0.72					1.310
QG1		0.65				1.547
QG2		0.702				1.720
QG3		0.703				1.684
QG4		0.762				1.705
QG5		0.71				1.517
QG6		0.771				1.896
QP1			0.735			1.576
QP2			0.763			1.739
QP3			0.698			1.436
QP4			0.753			1.636
QP5			0.756			1.614
RG1				0.75		1.808
RG2				0.742		1.677
RG3				0.737		1.711
RG4				0.652		1.375
RG5				0.785		1.957
RG6				0.698		1.551
RG7				0.702		1.595
ZK1					0.808	1.942
ZK2					0.839	2.187
ZK3					0.789	1.813
ZK4					0.808	1.851

### Path coefficient analysis

The Bootstrapping method was adopted to calculate the *T* statistic of each path coefficient, and the specific parameters were shown in Table 6 to test the significance level of the path coefficient estimate (two-tailed test). Among them, if 1.96 < *T* < 2.58, the path coefficient was significant at the 0.05 level; if 2.58 < *T* < 3.29, it was estimated to be significant at the 0.01 level; if *T* > 3.29, it was significant at the 0.001 level. The *T* statistic of the structural equation model in the Bootstrapping test showed that all path coefficients had high *T* statistic, and the *P*-value of each path was less than 0.05, indicating that each path coefficient passed the test of the corresponding significance level, and the model structure has high stability (Streukens and Leroi-Werelds, 2016).

**TABLE 6:** Model path coefficients.

(*M*)	Original sample (*O*)	Mean	Standard deviation (STDEV)	*T* statistic (|O/STDEV|)	*P*-value
Affective bias mediates → Cognitive bias	0.396	0.396	0.05	7.958	0
Affective empathy → Cognitive bias	0.311	0.306	0.069	4.509	0
Self-affirmation → Affective bias mediates	0.656	0.656	0.047	14.067	0
Self-affirmation → Affective empathy	0.757	0.756	0.042	17.832	0
Self-affirmation → Cognitive empathy	0.722	0.722	0.045	16.071	0
Cognitive empathy → Cognitive bias	0.206	0.213	0.078	2.652	0.008

From the model performance results, the hypothesis of H1a, H1b, H1c, H2a, H2b, and H2c has been verified in this study. In H1a, parents’ self-affirmation has a positive influence on emotional empathy, and the path coefficient is 0.757, indicating that parents will have emotional empathy for the Internet and teenagers’ online events under the influence of self-affirmation. In H1b, parents’ self-affirmation has a significant positive influence on cognitive empathy, and the path coefficient is 0.722, indicating that parents’ self-affirmation will affect their views and cognition toward the Internet. In H1c, self-esteem has a significant positive influence on the emotional deviation, and the path coefficient was 0.656, shows that parents’ sense of self-esteem tend to emotional events in the Internet and teenagers produce deviation, lead to a certain degree of prejudice on the Internet on the cognition of, but in contrast, this kind of emotional deviation is lower than shadow to emotion and cognition Ring. In H2a, the occurrence of paternal emotional empathy has an impact on the generation of cognitive bias and the path coefficient is 0.311. In H2b, the occurrence of paternal cognitive empathy has an impact on the generation of cognitive bias, and the path coefficient is 0.206. In H2b, the occurrence of paternal affective bias will affect the generation of cognitive bias, and the path coefficient is 0.396 in emotional empathy cognitive empathy. Among the three effects of affective bias on cognitive bias, affective bias has the most obvious effect on cognitive bias, indicating that when parents are emotionally impatient with the Internet and teenagers’ Internet use, their cognitive bias toward the Internet will be deeper and their prejudice against the Internet will be worst.

### Analysis of specific indirect effects

To verify the hypothesis of the proposed mediating relationship, the study utilized the Bootstrapping algorithm to examine the specific indirect effects, which randomly sampled 5,000 times. As for “Self-affirmation → Affective empathy → Cognitive bias,” the original sample (*O*) coefficient was 0.235, and the sample mean (*M*) coefficient was 0.232, the standard deviation (STDEV) coefficient was 0.055, the *T* statistic was 4.262, the *P*-value was 0.000; as for “Self-affirmation → Affective bias mediates → Pride and prejudice,” the original sample (*O*) coefficient was 0.260, the sample mean (*M*) coefficient was 0.260, the standard deviation (STDEV) coefficient was 0.039, the *T* statistic was 6.728, and the *P*-value was 0.000; as for “Self-affirmation → Cognitive empathy → Pride and prejudice,” the original sample (*O*) coefficient was 0.148, the sample mean (*M*) coefficient was 0.154, the standard deviation (STDEV) coefficient was 0.059, and the *T* statistic was 2.525, the *P*-value was 0.012. The test results in Table 7 show that the three mediated paths have significance *p*-values less than 0.05 at 95% confidence intervals that do not include the null point, indicating that this PLS-SEM is a fully mediated model and the hypothesis holds. Therefore, the hypothesis of H3a, H3b, and H3c in this study has been verified, the cognitive bias of parents toward the Internet under self-affirmation will be affected by the three empathic mediators of emotional empathy, cognitive empathy, affective bias, and the mediating effect of emotional empathy is slightly higher than that of cognitive empathy and affective bias.

**TABLE 7:** Table of test results for specific indirect effects.

	Original sample (*O*)	Mean (M)	Standard deviation (STDEV)	*T* statistic (|O/STDEV|)	*P*-value	Lower	Upper
Self-affirmation → Affective empathy → Cognitive bias	0.235	0.232	0.055	4.262	0	0.135	0.335
Self-affirmation → Affective bias mediates → Cognitive bias	0.26	0.26	0.039	6.728	0	0.186	0.338
Self-affirmation → Cognitive empathy → Cognitive bias	0.148	0.154	0.059	2.525	0.012	0.034	0.267

## Research conclusion

### Conclusion

Based on the exploration of the causes of adolescent Internet addiction and a systematic review of theories of self-affirmation, empathy, and cognitive biases, this paper analyzes the mechanisms underlying the generation of cognitive biases in parents’ perceptions of the Internet and adolescent Internet behavior in the modern Internet perspective. The study applies SmartPLS to validate the theoretical model proposed in this paper, and the results show that parents’ self-affirmation has a positive influence on the occurrence of empathy. Driven by a sense of self-affirmation, parents develop subjective affective and conscious tendencies toward the Internet and their adolescents’ access to the Internet. At the same time, the occurrence of empathic processes also enriched parents’ knowledge about the Internet and adolescents’ Internet access. In addition, the study also verified that parents’ self-affirming consciousness is an antecedent to the generation of cognitive biases and that this process is influenced by the mediation of empathy, suggesting that when parents’ sense of self-affirmation is stimulated by the mediation of empathy, it intensifies their cognitive biases. In the context of the rapid development of the Internet, the trend toward convenient Internet use is inevitable, but parents must go through rational analysis and judgment in their perception of the relationship between the Internet and their adolescents’ access to the Internet and establish a good parent–child relationship in order to promote parents’ and adolescents’ understanding of each other.

### Theoretical significance

The rapid development of the Internet has shifted the connection between adolescents and others from the real world to the virtual world, which also affects parent–child relationships. It is essential to explore the causes of adolescents’ Internet addiction and promote the stable development of parent–child relationships. Theoretically, the current study is of great significance. On the one hand, while reviewing the theories of self-affirmation, empathy, parent–child relationship and other related ones, this paper innovatively proposes the idea of cognitive bias, and explores the causes of adolescent Internet addiction from this perspective. In the study, we introduced the concept of empathy in psychology, which is innovative from previous studies focusing on family and social factors (Chen X. et al., 2020), and pointed out the existence of a parental habit of blind trust in self, inability to engage in dialectical rational thinking and excessive self-interest in families under the impact of Internet information, which broadened the research horizon as well. On the other hand, this study examines how self-affirmation influences Internet cognitive bias in parents under the condition of empathy as a mediating factor by cutting from a self-affirmation perspective, and examines the mediating utility of the empathy process, thus validating previous perspectives (Amiri and Jamali, 2019; Chen J. et al., 2020). It both enriches the research related to empathy and the causes of adolescent Internet addiction and provides a new perspective for other scholars to conduct related research in the future.

### Practical significance

While enjoying the convenience of the Internet, we should also pay attention to the impact of the Internet on the healthy growth of adolescents. This study explores the causes of adolescent Internet addiction from the perspective of parental self-affirmation with the following practical significance. First, the findings suggest that increased parental self-affirmation deepens parents’ cognitive bias toward the Internet and their adolescents’ access to the Internet. Therefore, in order to mitigate the effects of such cognitive bias, parents should actively change their inherent thinking about the Internet and view it from a more comprehensive and rational perspective; second, the study shows that the empathic relationship between parents and children affects the degree of cognitive bias of parents, and a good parent–child relationship can alleviate parents’ cognitive bias, which to a certain extent also reveals the necessity of maintaining a harmonious parent–child relationship; furthermore, the research has certain guiding significance for the family education of adolescents who are addicted to the Internet, and only by helping both parties to make efforts to build bridges of family communication and enhance their understanding of each other, can we create a good family education atmosphere for them.

### Limitations

This study has enriched the research on the causes of the adolescent Internet addiction, but there are still deficiencies in need of further exploration and improvement. First of all, the data collected in this study are of different qualities. The parents’ cognition of the Internet and their attitudes to the adolescents’ access to the Internet show heterogeneity under the influence of factors such as different family backgrounds, educational levels, and ideological concepts. Thus, future research can attempt to collect more high-quality, extensive and targeted data to further explore and verify the causes of the adolescent Internet addiction under the self-affirmation consciousness of the parents. Second, this study conducted the questionnaire survey to verify the research hypotheses one by one, however, as the data analyzed are horizontal, it is difficult to observe the dynamic process of the formation of cognitive bias against the Internet under the self-affirmation consciousness of the parents. Hence, longitudinal studies can be conducted in the future to further explore the formation rules and characteristics of the cognitive bias of the parents to provide feasible suggestions for the parents to establish a correct cognition in the Internet era and create a sound parent–child relationship for the healthy growth of the adolescents.

## Data availability statement

The original contributions presented in this study are included in the article/supplementary material, further inquiries can be directed to the corresponding author.

## Ethics statement

The studies involving human participants were reviewed and approved by Nanchang Institute Technology, China. The patients/participants provided their written informed consent to participate in this study. Written informed consent was obtained from the individual(s) for the publication of any potentially identifiable images or data included in this article.

## Author contributions

MZ contributed to the empirical work, analysis of the results, and writing of the first draft. JZ and HZ supported the total work of the MZ. ZZ contributed to overall quality and supervision the part of literature organization. GJ contributed to developing research hypotheses and revised the overall manuscript. All authors discussed the results and commented on the manuscript.
